# Rho-kinase/myosin light chain kinase pathway plays a key role in the impairment of bile canaliculi dynamics induced by cholestatic drugs

**DOI:** 10.1038/srep24709

**Published:** 2016-05-12

**Authors:** Ahmad Sharanek, Audrey Burban, Matthew Burbank, Rémy Le Guevel, Ruoya Li, André Guillouzo, Christiane Guguen-Guillouzo

**Affiliations:** 1 INSERM U991, Liver Metabolisms and Cancer, Rennes, France; 2 Rennes 1 University, Rennes, France; 3 ImPACcell platform, Biosit, Rennes 1 University, Rennes, France; 4 Biopredic International, St Grégoire, France

## Abstract

Intrahepatic cholestasis represents a frequent manifestation of drug-induced liver injury; however, the mechanisms underlying such injuries are poorly understood. In this study of human HepaRG and primary hepatocytes, we found that bile canaliculi (BC) underwent spontaneous contractions, which are essential for bile acid (BA) efflux and require alternations in myosin light chain (MLC2) phosphorylation/dephosphorylation. Short exposure to 6 cholestatic compounds revealed that BC constriction and dilation were associated with disruptions in the ROCK/MLCK/myosin pathway. At the studied concentrations, cyclosporine A and chlorpromazine induced early ROCK activity, resulting in permanent MLC2 phosphorylation and BC constriction. However, fasudil reduced ROCK activity and caused rapid, substantial and permanent MLC2 dephosphorylation, leading to BC dilation. The remaining compounds (1-naphthyl isothiocyanate, deoxycholic acid and bosentan) caused BC dilation without modulating ROCK activity, although they were associated with a steady decrease in MLC2 phosphorylation via MLCK. These changes were associated with a common loss of BC contractions and failure of BA clearance. These results provide the first demonstration that cholestatic drugs alter BC dynamics by targeting the ROCK/MLCK pathway; in addition, they highlight new insights into the mechanisms underlying bile flow failure and can be used to identify new predictive biomarkers of drug-induced cholestasis.

Intrahepatic cholestasis represents a frequent manifestation of drug-induced liver injury (DILI) in humans^1^. In several population-based studies of DILI, a cholestatic pattern and a mixed pattern were observed in 20–40% and 12–20% of the patients, respectively. The mortality rate in patients has been estimated as 7.8% in certain studies, although it can be lower (2.5%) in groups of patients with mixed hepatocellular and cholestatic dysfunction^2^. However, the primary problem associated with cholestasis is that accurately predicting its risk is extremely difficult^3^.

A frequently reported causal mechanism underlying cholestatic disease is hepatobiliary transporter system alterations, in particular alterations to the bile salt export pump (BSEP/ABC11), which is the most physiologically important canalicular bile transporter^4^. Bile acid (BA) transport and secretion can also be impaired by the inhibition of BA uptake and efflux across the sinusoidal membrane. Although many cholestatic drugs are known to inhibit BSEP, several others are ineffective^5^. Therefore, the low prediction rate of the disease suggests that drug-induced cholestasis is linked to prior intracellular events involving one or more signalling pathway(s) that remain to be identified.

Membrane transporter efficiency, intracellular trafficking and efflux dynamics are necessarily interconnected via complex mechanisms that converge in dynamic movements that control bile clearance and involve signalling mechanisms that interfere with acto-myosin interactions. Rho GTPases play a critical role in actin distribution, which affects cytoskeleton organization and cell motility^6^. The RhoA/Rho-kinase (ROCK) pathway plays a major role in vasocontraction and vascular tone regulation^7^. Activation of the RhoA/ROCK pathway is also essential for the contraction of vascular smooth muscle^8^.

The first step for activating the RhoA/ROCK pathway involves G protein-coupled vasopressor receptors and contractile agonists. These receptors activate the small monomeric GTPase RhoA, which activates ROCK and leads to MYPT1 phosphorylation and myosin light chain phosphatase (MLCP) inhibition, thereby resulting in the enhanced phosphorylation of myosin light chain (MLC). This phosphorylation catalyses interactions between the myosin head and actin and subsequently allows myosin ATPase to produce a sliding force that results in acto-myosin contraction^9^ (Fig. 1). Myosin II contractile activity in smooth muscle and non-muscle cells is also stimulated through the phosphorylation of MLC by Ca^2+^/calmodulin (CaM)-dependent myosin light-chain kinase (MLCK)^10^.

Signalling pathways have been identified as fundamental mechanisms that control bile canaliculi (BC) formation. Previous studies have shown that the ROCK pathway plays a major role in establishing bile ductular polarity in hepatic cells^11^, whereas other studies have demonstrated that BAs stimulate canalicular network formation and maintenance via the cAMP-liver kinase-B1 and AMP kinase-dependent pathways by affecting the actin cytoskeleton via phosphorylation of MLC2 and tight junction assembly. This process occurs directly or indirectly through small GTPases, which alter the cellular energy status^12^,^13^,^14^. ROCK was also found to mediate the regulation of intrahepatic vascular tone in humans with cirrhotic livers and in rats with bile ductular ligation^15^. However, a direct contribution of the ROCK/MLCK pathway to intrahepatic BC disorders has not been described.

Polarized hepatocytes are essential for bile flow, and a loss of polarity causes bile secretory failure and cholestasis^16^. Primary human hepatocyte cultures, particularly in a collagen sandwich configuration (SCHH), form a multicellular canalicular network as existing *in vivo*^17^. The differentiated human HepaRG cell line, which expresses phase 1 and 2 drug metabolizing enzymes and transporters and forms polarized structures with functional BC, was successfully used in the *in vitro* production of BAs that mimicked features of intrahepatic cholestasis induced by chlorpromazine (CPZ) and cyclosporine A (CsA) treatment and the characterization of mechanisms involved in the initiation of lesions^18^,^19^,^20^.

In this study, 6 compounds with diverse chemical structures were selected, and they all have the ability to induce BC deformations (Supplementary Table 1). These compounds include the cholestatic drugs CPZ, CsA and bosentan; the hepatotoxicant α–naphthyl isothiocyanate (ANIT), which is largely used in rodent models of human intrahepatic cholestasis and biliary function disruption, although the underlying mechanism remains unclear^21^; the Y-27632 ROCK inhibitor analogue fasudil, which is used in combination with bosentan for the treatment of pulmonary arterial hypertension; and the secondary BA deoxycholate (DCA). A previous study showed that DCA infusion in rats resulted in canalicular membrane structural alterations accompanied by reduced excretory functions in the liver^22^. Using these cholestatic agents and taking advantage of the well-polarized HepaRG and human hepatocytes, we investigated whether the ROCK/MLCK pathway has a critical role in cytoskeleton rearrangement and the BC deformations that accompany cholestatic insults.

## Results

### Morphological alterations of BC induced by the tested compounds

**Figure 2:**
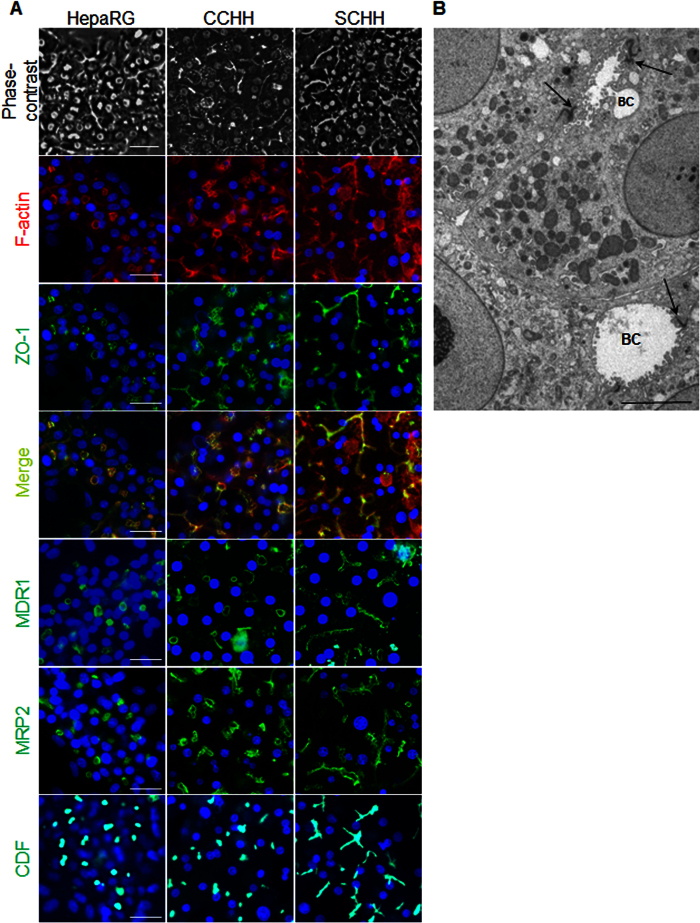
Polarity of human HepaRG and primary hepatocytes. (**A**) Phase-contrast microscopy examination of the BC in HepaRG hepatocytes, CCHH and SCHH. Rhodamine-phalloidin fluoroprobe staining of pericanalicular F-actin (red); immunolocalization of the junctional ZO-1 protein (green). Merged images showing that the junctional ZO-1 protein (green) co-localizes with the pericanalicular F-actin (red) in HepaRG cells CCHH and SCHH. Immunolocalization of the hepatobiliary transporters MDR1 and MRP2 (green). CDF accumulation in BC (green). Hoechst-labelled nuclei (blue). Fluorescence images were obtained with a Cellomics ArrayScan VTI HCS Reader (bar = 30 μm). (**B**) Electron microscopy examination of the tight junctions surrounding BC in HepaRG cells (arrows) (bar = 10 μm).

Phase-contrast examinations and rhodamine-phalloidin fluoroprobe labelling of cytoskeletal F-actin showed that untreated differentiated HepaRG cells as well as conventional cultured human hepatocytes (CCHH) have large biliary pockets (saccular lumen: S-BC) that branch out to smaller ductules (tubular lumen; T-BC) that usually occur in one extremity. In SCHH, however, most ductules are in a tubular form (T-BC) and form a network of connections. BC integrity was visualized by immunolocalization of the junctional zona occludens-1 protein (ZO-1), which co-localized with pericanalicular F-actin (Fig. 2A). Canalicular transporters, multidrug resistance protein 1 (MDR1/ABCB1) and multidrug-resistance-related protein 2 (MRP2/ABCC2), were correctly distributed on across the canalicular membranes. The accumulation of 5 (and 6)-carboxy-2′,7′-dichlorofluorescein (CDF) into the BC lumen confirmed the integrity as well as the activity up to the canalicular poles (Fig. 2A). Typical BC of different sizes closed by tight junctions were also observed under an electron microscope (Fig. 2B). These characteristics were similar in the three cell models (HepaRG cells, CCHH and SCHH).

**Figure 3:**
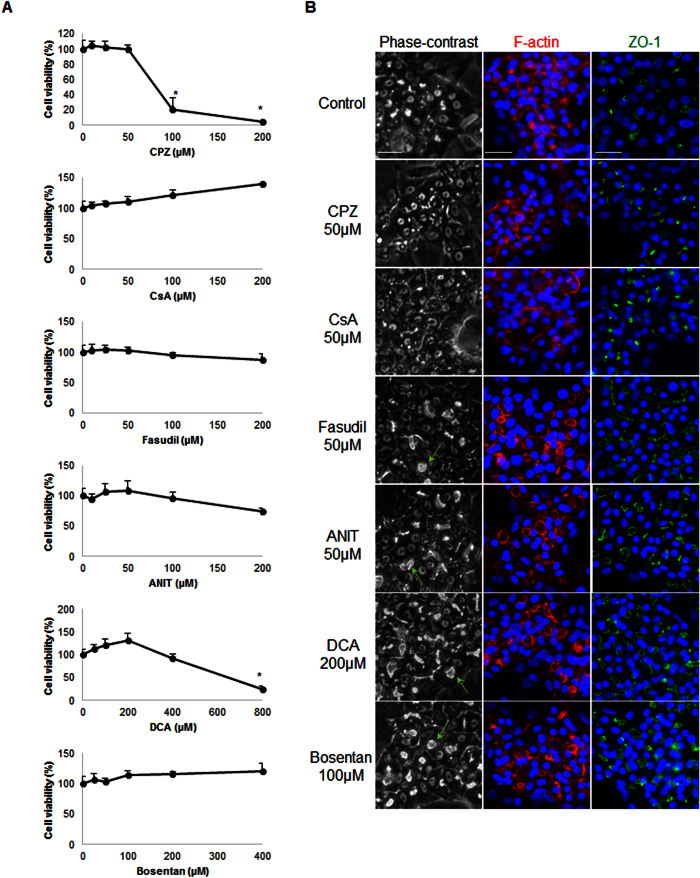
Cytotoxicity evaluation and alteration of the BC morphology by the tested compounds in HepaRG cells. (**A**) Cells were incubated for 4 h with different concentrations of CPZ (0–200 μM), CsA (0–200 μM), fasudil (0–200 μM), ANIT (0–200 μM), DCA (0–800 μM) and bosentan (0–400 μM). Cytotoxicity was measured by the MTT colorimetric assay. Data were expressed relative to the untreated cells, which were arbitrarily set at a value of 100%. Data represent the means ± SEM of three independent experiments. (**B**) Untreated cells and cells treated with 50 μM CPZ, 50 μM CsA, 50 μM fasudil, 50 μM ANIT, 200 μM DCA or 100 μM bosentan. Phase-contrast images were captured after 3 h; BC (arrows); F-actin localized using rhodamine-phalloidin fluoroprobe (red). Immunolabelling of the junctional ZO-1 protein (green) in HepaRG cells treated with the tested compounds compared with that of the control cells. Nuclei stained in blue (Hoechst dye). The images were obtained with a Cellomics ArrayScan VTI HCS Reader (bar = 30 μm).

**Figure 4:**
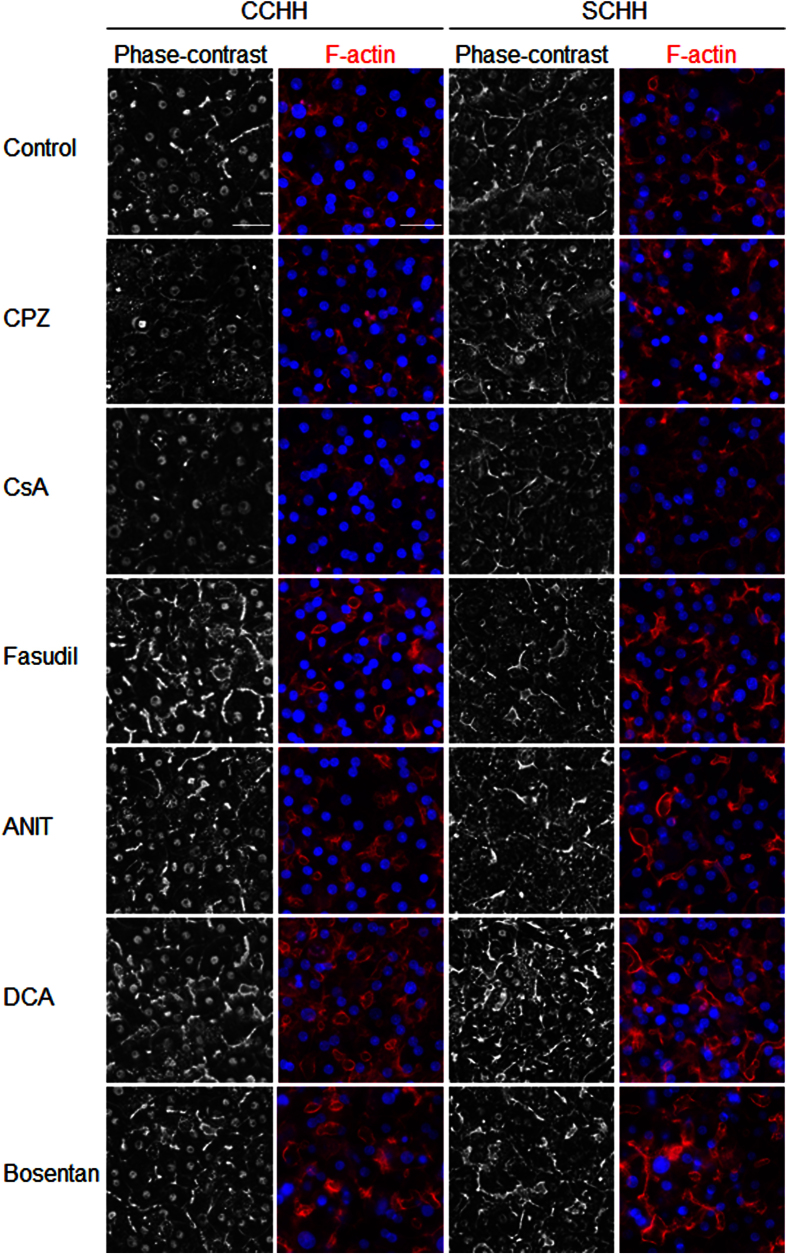
Alteration of the BC morphology by the tested compounds in CCHH and SCHH. Untreated cells and cells treated with 50 μM CPZ, 50 μM CsA, 50 μM fasudil, 50 μM ANIT, 200 μM DCA, or 100 μM bosentan. Phase-contrast images were captured after 3 h. F-actin was localized using a rhodamine-phalloidin fluoroprobe (red). Nuclei stained in blue (Hoechst dye). (bar = 30 μm).

A short exposure to the 6 cholestatic compounds was used to investigate the early events that led to BC deformations in the three cell models. The toxicity of the 6 compounds was evaluated in HepaRG cells using the MTT test after a 4 h exposure (Fig. 3A). Non-toxic concentrations (50 μM each) of CPZ, CsA, fasudil and ANIT; 200 μM DCA; and 100 μM bosentan deformed most S-BC structures (Figs 3 and 4). Therefore, these concentrations were selected for further analysis. Phase-contrast imaging showed that exposure to CPZ or CsA resulted in the progressive constriction of S-BC, whereas exposure to the other 4 compounds (i.e., fasudil, ANIT, DCA and bosentan) resulted in spectacular dilation of S-BC in both human HepaRG and primary hepatocytes. S-BC deformations were confirmed by rhodamine-phalloidin staining of pericanalicular F-actin (Figs 3B and 4) and localization of the junctional ZO-1 protein in HepaRG cells (Fig. 3B).

### Associated dysfunctions of BC dynamics

**Figure 5:**
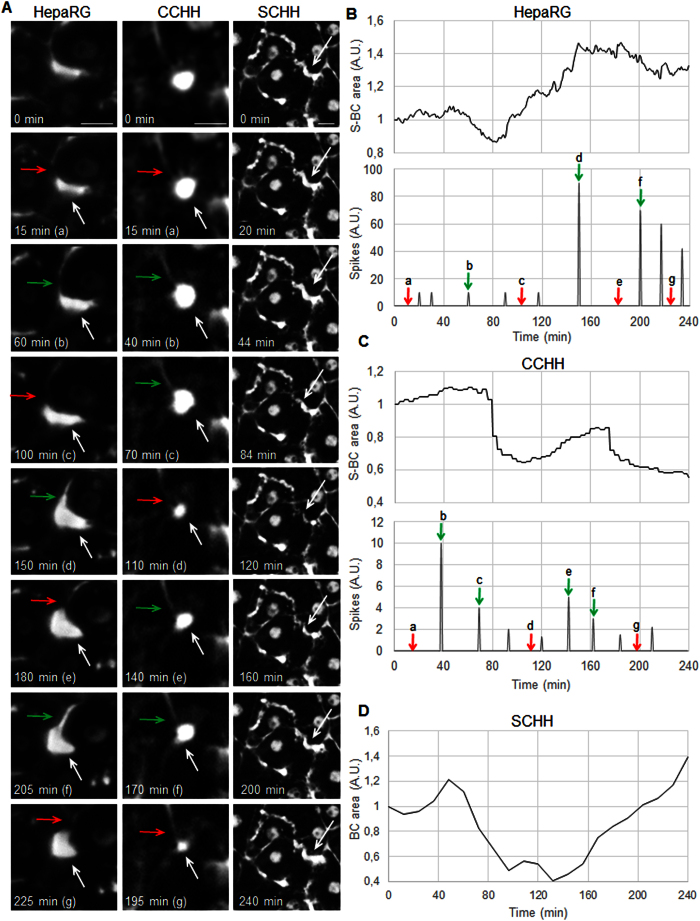
Dynamics of BC in human HepaRG and primary hepatocytes. (**A**) Time-lapse imaging showing unidirectional rhythmic opening (green arrows) and closing (red arrows) spikes associated with contraction/relaxation of S-BC (white arrows). (**B–D**) Quantification of S-BC area in arbitrary units (A.U.) and graphic representation of spikes. Imaging was performed using an inverted microscope (bar = 10 μm), and video analysis was performed using a modelling tool (Tracker 4.87).

Using time-lapse microscopy, the BC of untreated HepaRG hepatocytes revealed spontaneous rhythmic motility characterized by repeated opening and closing spikes every 20–30 min (Fig. 5A; Supplementary video 1). These spikes allowed for the dynamic evacuation of products from the S-BC into the T-BC, which led to reductions in the S-BC lumen size (Fig. 5B). Comparisons with normal CCHH showed similar rhythmic movements (Fig. 5A–C). T-BC underwent contraction/relaxation cycles with no detectable spikes, which may have been related to the tubular nature of BC and the scarcity of S-BC in SCHH (Fig. 5A–D; Supplementary videos 2 and 3).

**Figure 6:**
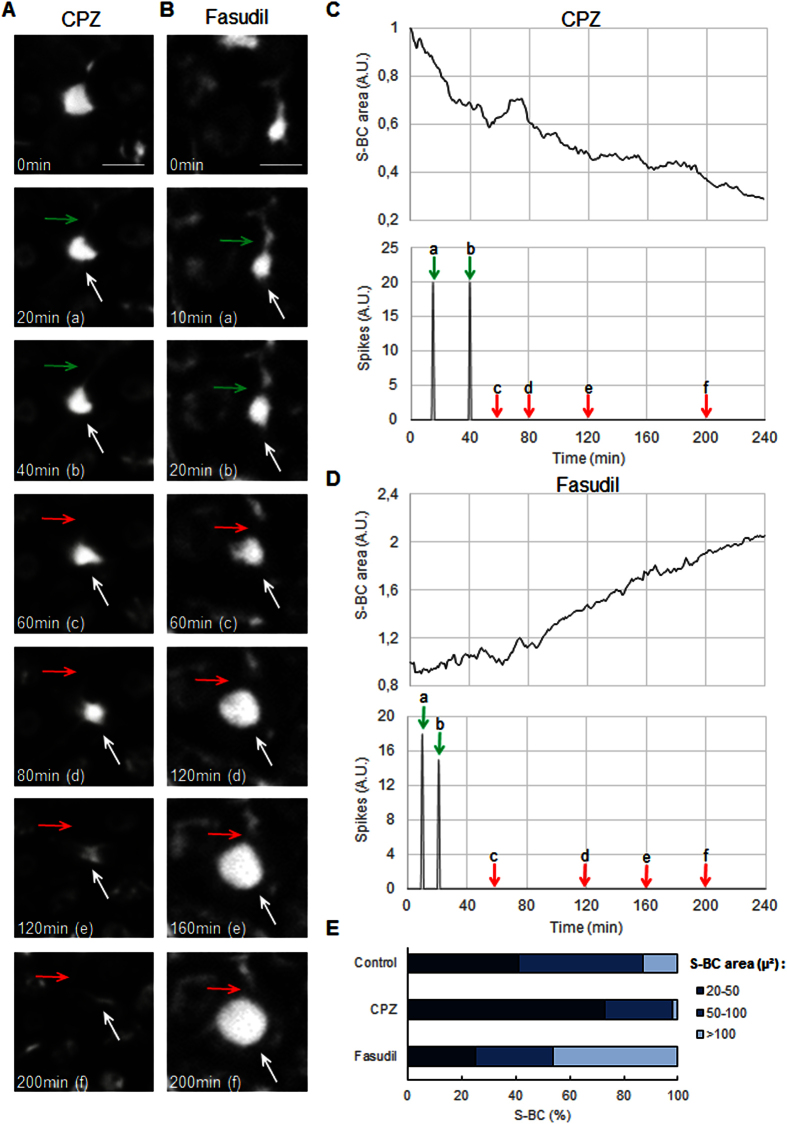
Disruption of BC rhythmic movements in CPZ- and fasudil-treated HepaRG hepatocytes. (**A,B**) Representative time-lapse imaging of HepaRG cells treated with 50 μM CPZ or 50 μM fasudil for 4 h (bar = 10 μm). (**C**,**D**) Quantification of the S-BC area and spikes showing the early disappearance of rhythmic spikes (green/red arrows) with both drugs. Occurrence of permanent constriction of S-BC with CPZ and dilation with fasudil (white arrows). (**E**) Quantification of the BC area based on ZO-1 protein distribution (as in Fig. 3) using Cellomics ArrayScan VTI HCS Reader software as described in the Materials and Methods. The BC were grouped into 3 categories according to their area.

We further analysed the spike rhythms in the presence of the different compounds. For instance, within 1 h of the HepaRG cell treatment, CPZ induced permanent constriction of the S-BC, whereas fasudil had induced strong dilation (Fig. 6A,B). However, both treatments led to a static state of S-BC that was characterized by the total disappearance of spikes and a loss of connections between the S-BC and T-BC (Fig. 6A–D, Supplementary videos 4 and 5). The ZO-1 protein distribution was used to measure the BC areas. For example, 73% of the S-BC from the cell layers exposed to CPZ had lumen with a size ≤50 μm^2^ compared with 41% of the S-BC in untreated cells. However, in the presence of fasudil, up to 46% of the S-BC were ≥100 μm^2^ compared with only 13% in untreated cultures (Fig. 6E). These results indicated that alterations occurred in the majority of BC in HepaRG hepatocytes treated with cholestatic drugs.

### Alteration of bile flow as a consequence of BC dynamic disorders

**Figure 7:**
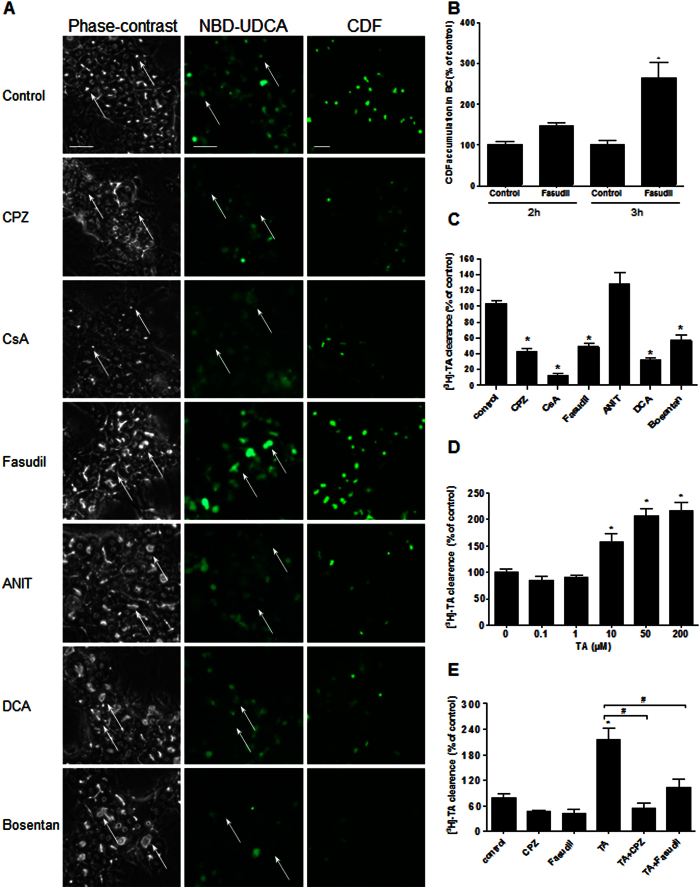
Effects of the tested compounds on BA clearance. (**A**) NBD-UDCA and CDF efflux. White arrows indicate fluorescence in the BC (bar = 30 μm). (**B**) Quantification of CDF accumulation in the BC after fasudil treatment. (**C,D**) [^3^H]-TA clearance in the HepaRG cells treated for 2 h with either the tested compounds or different concentrations of unlabelled TA. (**E**) [^3^H]-TA clearance in the cells treated with either CPZ or fasudil or co-treated with unlabelled TA. The data were expressed relative to that of the untreated cells arbitrarily set at 100%. The data represent the means ± SEM of 3 independent experiments. *p < 0.05 compared with that of the untreated cells, ^#^p < 0.05 compared with that of TA (50 μM) alone.

To demonstrate that contractile dynamic movements have serious consequences on bile flow activity, we used two labelled BAs, 3α-hydroxy-7-nitrobenzoxadiazolyl-ursodeoxycholic acid (NBD-UDCA) fluorescent analogue and [^3^H]-TA, as well as CDFDA. When the control and treated HepaRG cells were exposed to NBD-UDCA or CDFDA, the effects of the compounds on BA intracellular trafficking and efflux at the canalicular lumen could be detected. After incubation, fluorescent NBD-UDCA and CDF were found in the BC of untreated cells, whereas they were barely detected in the constricted BC of CPZ- and CsA-treated cells or in the large isolated BC cisternae induced by ANIT, DCA and bosentan. However, both fluorescent substrates accumulated in the isolated BC cisternae of fasudil-treated cells, although the clearance of BC was delayed compared with that of untreated cells (Fig. 7A). Indeed, a 2.7-fold higher number of CDF-positive BC were observed within 3 h in the fasudil-treated cells compared with that in the corresponding control cells (Fig. 7A,B). These results indicate a major failure in bile flow that was correlated with an abnormal morphology of BC and a loss of their dynamic movement induced by all of the cholestatic compounds.

All of the tested compounds induced an accumulation of [^3^H]-TA in the cell layers. A reduction in BA clearance to 43% (p < 0.0001), 13% (p < 0.0001), 49% (p = 0.0002), 32% (p < 0.0001) and 57% (p = 0.001) was observed with CPZ, CsA, fasudil, DCA and bosentan, respectively. Importantly, accumulations were not detected after the ANIT treatment (Fig. 7C).

A previous study^23^ showed that the addition of TA accelerated the frequency of BC contractions compared with that of untreated cells. Consistent with the increased dynamics of BC, TA loading increased the clearance of [^3^H]-TA in a dose-dependent manner to 157% (p = 0.02), 207% (p < 0.0001) and 216% (p < 0.0001) at 10, 50 and 100 μM, respectively (Fig. 7D). Interestingly, the treatment with 50 μM CPZ and 50 μM fasudil lowered the TA-induced increase in [^3^H]-TA clearance to 55% (p = 0.0004) and 103% (p = 0.01), respectively, whereas the 50 μM TA treatment presented a clearance of 215% (Fig. 7E).

### Drug-induced bile flow failure is associated with the modulation of the ROCK pathway

**Figure 8:**
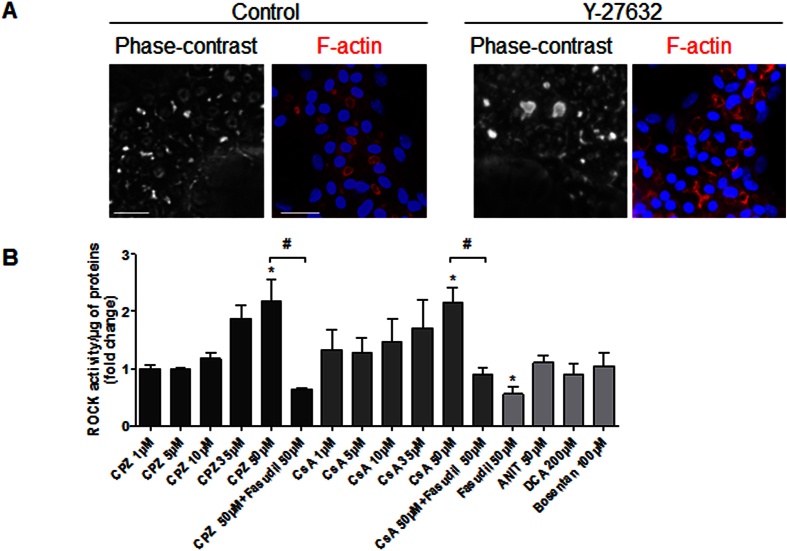
Alteration of ROCK activity by the tested compounds. (**A**) Untreated and 20 μM Y-27632-treated cells. Phase-contrast images were captured after 3 h. F-actin was localized using a rhodamine-phalloidin fluoroprobe (red). Hoechst-labelled nuclei are shown in blue (bar = 30 μm). Note the BC dilation in the presence of Y-27632 cells. (**B**) ROCK activity after a 1-h treatment of HepaRG cells using a ROCK activity assay Kit (Millipore, catalogue CSA001). The data were expressed relative to that of the untreated cells and are represented as the means ± SEM of 3 independent experiments. *p < 0.05 compared with that of the untreated cells, ^#^p < 0.05 compared with that of either CPZ (50 μM) or CsA (50 μM) alone.

ROCK has pleiotropic functions and primarily regulates cellular contraction, motility, morphology and polarity. We examined potential links between disorders of BC dynamics induced by the tested compounds and the ROCK pathway. Interestingly, BC dilation was observed in the presence of Y-27632 (20 μM), a specific ROCK inhibitor, indicating a role of ROCK in controlling the morphology of BC (Fig. 8A). Next, we analysed the effects of the tested compounds on ROCK activity after 1 h of treatment. At low concentrations, CPZ and CsA did not significantly modify ROCK activity, whereas both compounds induced up to 2-fold increases of ROCK activity at doses that caused a constriction of BC. However, fasudil inhibited ROCK activity by 50% (p = 0.01) and caused a strong dilation of BC, which was expected. No significant ROCK inhibition was observed with ANIT, DCA and bosentan (Fig. 8B).

### Drug-induced bile flow failure is associated with altered myosin II activation

**Figure 9:**
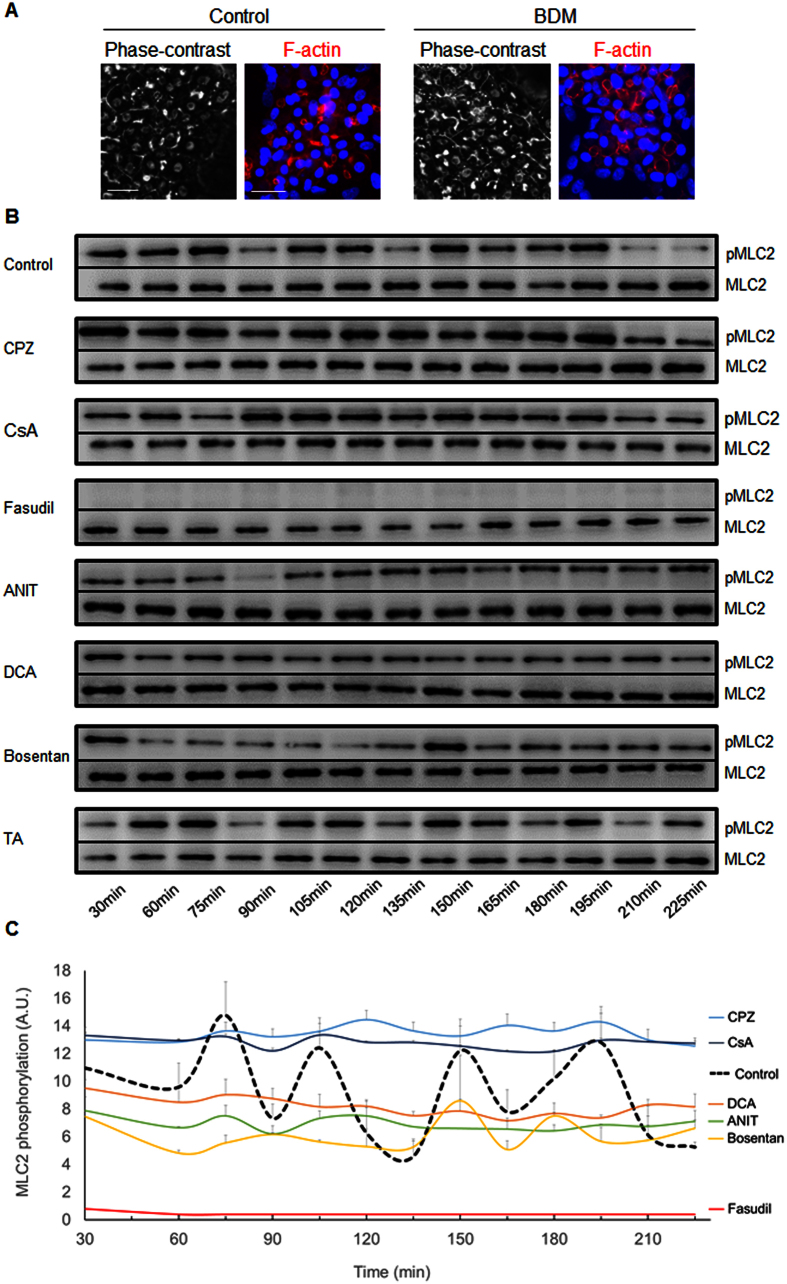
Alteration of MLC2 phosphorylation/dephosphorylation rhythms by the tested compounds. (**A**) Untreated and 20 mM BDM-treated HepaRG cells. Phase-contrast images were captured after 3 h. F-actin was localized using a rhodamine-phalloidin fluoroprobe (red). Hoechst-labelled nuclei are shown in blue (bar = 30 μm). (**B**) Representative western blots of the p-MLC2/total MLC2 forms at various time points. (**C**) Graphic representation of MLC2 phosphorylation/dephosphorylation quantified using ImageJ 1.48 software. The data were expressed in arbitrary units (A.U.) and represented as the means ± SEM of 3 independent experiments.

Non-muscle myosin II is an actin-based motor protein that is controlled by ROCK. Phosphorylation of MLC2, the regulatory subunit of myosin II, at serine-19 results in increased myosin ATPase activity and acto-myosin contractility^24^. We postulated that the dynamic movements of BC could be controlled by pericanalicular myosin activity. First, we used the myosin heavy chain ATPase inhibitor BDM (20 mM) and observed alterations of BC that led to their dilation, which was also observed with fasudil, ANIT, DCA, and bosentan (Fig. 9A). Further analysis of the phosphorylation state of MLC2 in the untreated cells demonstrated alternating regular phases of phosphorylation and dephosphorylation of MLC2 every 30–45 min, which were coordinated with BC spikes, indicating a role for myosin II in controlling BC dynamics (Fig. 9B,C). Treatment with the 6 tested compounds resulted in an important disruption of the MLC2 phosphorylation/dephosphorylation rhythm. Treatment with CPZ and CsA induced permanent MLC2 phosphorylation and led to permanent constriction of the S-BC. However, fasudil drastically and permanently inhibited MLC2 phosphorylation and caused strong dilation of the BC. This phosphorylation/dephosphorylation rhythm was also inhibited (although to a lesser extent) by ANIT, DCA and bosentan (Fig. 9C). A 2-fold increase in the MLC2 phosphorylation/dephosphorylation frequency was observed in the presence of TA, which was correlated with the increased S-BC contraction/relaxation frequency described above (Fig. 9B).

### Both ROCK and MLCK are signalling targets of cholestatic compounds

**Figure 10:**
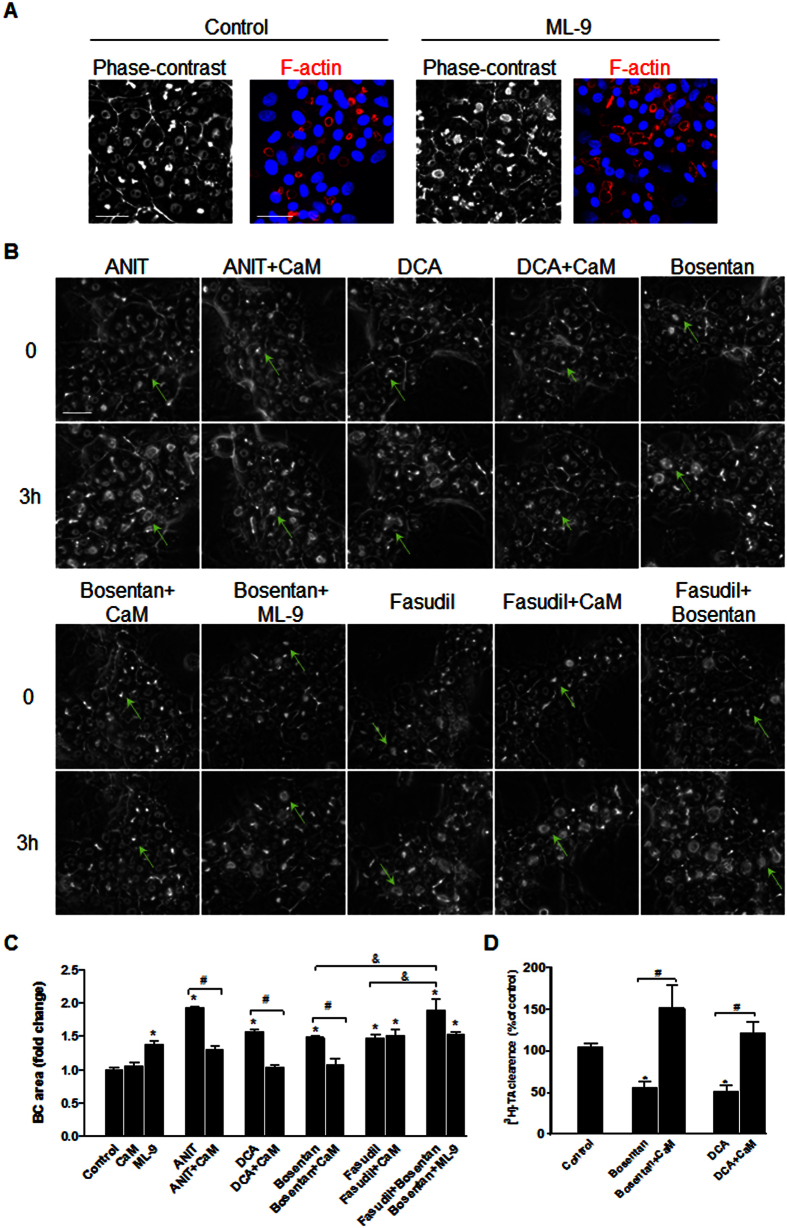
Alteration of the MLCK pathway with the tested compounds. (**A**) HepaRG cells exposed to 20 μM ML-9 for 3 h and the controls: phase-contrast microscopy and F-actin localization (bar = 30 μm). Note the BC dilation in the presence of ML-9 cells. (**B**) Time-lapse imaging of HepaRG cells treated with ANIT, DCA, fasudil or bosentan alone, co-treated with 5 μM CaM; or treated with bosentan + ML-9 or fasudil (bar = 30 μm); BC: green arrows. (**C**) Quantification of the BC area using ImageJ 1.48 software as described in the Materials and Methods. The data were expressed as the fold change in the BC mean area after 3 h relative to the mean area at T0 (means ± SEM of 3 independent experiments). *p < 0.05 compared with that of the untreated cells, ^#^p < 0.05 compared with that of the cultures treated with either ANIT, DCA or bosentan alone. ^&^p < 0.05 compared with that of the cultures treated with both bosentan and fasudil. (**D**) [^3^H]-TA clearance in HepaRG cells treated with bosentan and DCA alone or co-treated with CaM for 2 h. The data were expressed relative to that of the untreated cells arbitrarily set at 100% and are presented as the means ± SEM of 3 independent experiments. *p < 0.05 compared with that of the untreated cells, ^#^p < 0.05 compared with that of the cultures treated with either bosentan or DCA alone.

Because MLCK is coupled to ROCK and shares MLC2 as a common substrate, we evaluated whether MLCK also contributed to BC lumen alterations. In the presence of ML-9 (20 μM), a specific MLCK inhibitor, S-BC dilation was observed (Fig. 10A). Therefore, we hypothesized that although ANIT, DCA and bosentan do not have an effect on ROCK activity, they could act through MLCK inhibition. ANIT, DCA and bosentan alone increased the BC mean area by 1.9 (p < 0.0001), 1.56 (p = 0.0001) and 1.48 fold (p = 0.002) after 3 h of exposure, respectively. Co-treatment with calmodulin (CaM), a primary MLCK activator, counteracted the BC dilation induced by the 3 compounds, and CaM completely prevented the inhibition of [^3^H]-TA clearance induced by bosentan and DCA (Fig. 10D). Moreover, co-treatment with bosentan and ML-9 did not present an additive effect on BC dilation, indicating that the two compounds exerted their effect via the same MLCK enzyme target. However, CaM did not exert a protective effect against fasudil-induced dilation, indicating that fasudil did not act via MLCK, whereas bosentan combined with fasudil had an additive effect on BC dilation and resulted in a 1.89-fold (p = 0.01) increase in the mean area compared with the 1.48-fold and 1.47-fold increases with bosentan and fasudil alone, respectively. These results indicate that these compounds acted via distinct MLCK and ROCK enzymatic targets (Fig. 10B,C).

## Discussion

Cholestasis is a complex multifactorial disease with distinct characteristics. However, its main feature is its frequent association with abnormal BA elimination. Many authors have attempted to predict drug-induced cholestasis based on direct inhibition of the major canalicular BA transporters. Unfortunately, this approach is poorly predictive because many cholestatic drugs do not directly interfere with BA canalicular efflux transporters^25^,^26^,^27^,^28^. In the current work, we took advantage of metabolically competent human HepaRG and primary hepatocyte cells to demonstrate that drug-induced cholestasis was strongly associated with the deformation and motility failure of BC. In addition, we provided the first demonstration that the ROCK/MLCK pathway plays a critical role in disrupting BC dynamic movements via cholestatic agents.

As observed in the liver *in vivo*^29^, we found that the biliary saccular lumen and tubular canaliculi structures were reformed in normal cultured hepatocyte monolayers, with biliary saccular lumen primarily occurring at bifurcations in the bile canalicular structures of HepaRG cells and CCHH and tubular BC occurring more frequently in the primary hepatocytes maintained in a sandwich configuration. Moreover, the accumulation of F-actin fibres and specific localization of transporters and junctional proteins contributed to qualifying these characteristically polarized BC in both *in vitro* liver cell models^30^,^31^.

Biliary secretion is a complex process involving several steps, which include translocating BA across the basolateral membrane and then trafficking through the cytoplasm, transporting across the canalicular membrane, and effluxing out of the canalicular lumen. The motility of BC was mainly demonstrated in isolated hepatocyte couplets^32^ and has been reported to play a role in bile flow in rat hepatocytes both *in vitro* and *in vivo*^33^. For instance, it has been shown that interference in acto-myosin interactions by phalloidin, cytochalasin or lithocholic acid decreases the rate of spontaneous contractions of BC and reduces the flow of bile^34^,^35^,^36^. Herein, time-lapse microscopy was used to study human HepaRG and primary hepatocytes, and we observed contractions of BC characterized by repeated opening and closing processes as well as the unidirectional expulsion of what appeared to be bile products from the S-BC to a proximal small T-BC. Importantly, the contractions appeared to be essential for the clearance of BAs. TA loading resulted in an increased frequency of BC contractions accompanied by the increased efflux activity of TA, which is a strong evidence that these contractions have a role in bile flow^23^. The electron microscopy observations of neighbouring BC with different sizes also supported the occurrence of BC contractions.

One important observation of the present work was that BC motile activity was rapidly inhibited by exposure to various cholestatic compounds. By performing a comparative analysis of the 6 compounds, our study showed that BC could be deformed early depending on the tested compounds. Thus, for the first time, two main classes of cholestatic drugs could be distinguished, one that includes CPZ and CsA and induces canalicular lumen constriction and another that includes fasudil, ANIT, DCA and bosentan and induces canalicular lumen dilation. Interestingly, both classes of cholestatic drugs were associated with strong impairments of contractile movements. Under similar experimental conditions, the two non-cholestatic hepatotoxic drugs paracetamol and amiodarone did not cause obvious BC alterations (not shown). Noticeably, BC constriction as observed with CPZ and CsA might represent severe effects related to the induction of oxidative and endoplasmic reticulum stress at high drug concentrations, which ultimately lead to cell death as previously reported^18^,^19^. Further investigations of a large series of cholestatic and non-cholestatic drugs are needed to identify the mechanisms underlying these irreversible effects.

A failure of bile secretion fundamentally defines cholestatic disease and is associated with morphological and contractile disorders. Three main observations support the occurrence of bile secretion failure. i) Abnormal intracellular accumulation of [^3^H]-TA in the presence of CPZ and CsA as well as with fasudil, DCA and bosentan. This accumulation was dose-dependent regardless of whether these drugs constricted or dilated the canalicular lumen, and similar results have been reported with CPZ and CsA^18^,^19^,^20^, while this is the first description of the adverse outcome induced by the widely-used drug fasudil. ii) Concomitant markedly diminishment of BA clearance as indicated by the efflux of fluorescent NBD-UDCA in the presence of CPZ and fasudil. However, these deformed BC (constricted or dilated) appeared as inert cisternae without the ability to undergo dynamic contractions and opening in a rhythmic manner to discharge their contents, which was observed in the control BC. This result supports the hypothesis that impaired contractile movements of canalicular lumens are critical for clearance deficiencies. iii) Impairment of the compensatory feedback control of BA clearance in the presence of cholestatic drugs, which was shown with TA loading.

Because the functional basis of BC contraction/relaxation dynamics is mediated by actin filaments, cholestatic drugs would be expected to alter contractile movements, and this led us to identify the molecular mechanisms controlling myosin ATPase activity, which is a key step in the development of acto-myosin-based contractile forces. Motor myosin II activity is mainly controlled by phosphorylation of its light chain. Therefore, we investigated the two classes of enzymes, MLC kinases and Rho-kinases, which are known to be the major kinases that phosphorylate MLC *in vitro* and *in vivo*^37^,^38^. This work provides the first evidence that the ROCK/MLCK pathway and its associated enzymes are major targets of the cholestatic compounds that lead to deformation of the apical lumen and impairment of contractile movements. This conclusion is supported by the following evidence: (i) time-dependent alternations of MLC2 phosphorylation/dephosphorylation states in untreated cells were coordinated with the contraction frequency of BC; (ii) TA treatment increased the frequency of BC contractions and MLC2 phosphorylation/dephosphorylation and also increased the clearance of radiolabelled [^3^H]-TA; (iii) activation of ROCK activity was observed as early as 1 h after exposure to either CsA or CPZ and maintained an abnormally high MLC2 phosphorylation level; (iv) prevention of CsA- and CPZ-induced activation of ROCK by fasudil; (v) rapid, drastic and permanent MLC2 dephosphorylation leading to BC dilation by the stable and potent ROCK inhibitor fasudil; (vi) reduction of MLC2 phosphorylation by DCA, ANIT and bosentan, which was similar to that of fasudil although to a lower extent, and abrogation of the phosphorylation/dephosphorylation dynamics by targeting Ca^2+^/CaM-dependent MLCK instead of ROCK; and (vii) regulation of BC by actin via a contribution of the myosin II heavy chain ATPase as indicated by the effect of BDM, an inhibitor of this enzyme, which clearly mimicked MLCK and ROCK inhibitors by inducing BC dilation, thereby making this enzyme another potential signalling target for cholestatic compounds.

**Figure 11:**
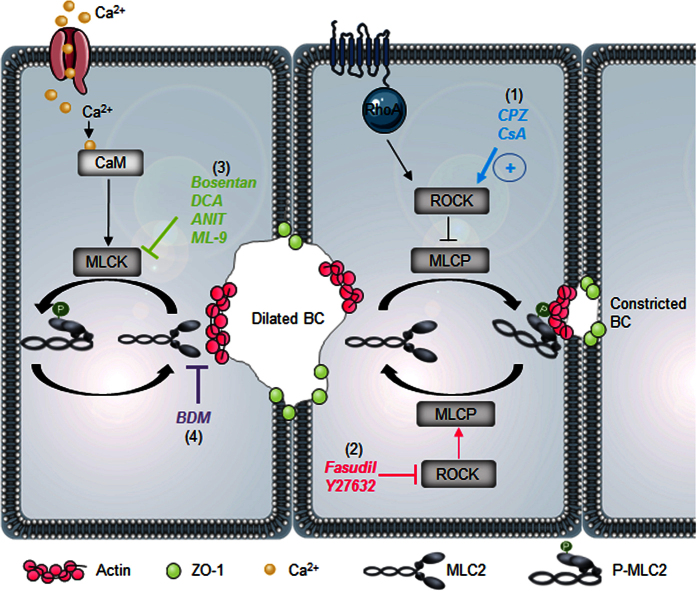
Schematic representation of the main molecular targets of the tested cholestatic compounds in the ROCK/MLCK pathway. (**1**) CsA and CPZ induced the activation of ROCK activity and maintained abnormally high MLC2, thereby leading to BC constriction. However, (**2**) fasudil and Y-27632 inhibited ROCK activity and caused MLC2 dephosphorylation, thereby leading to BC dilation. (**3**) DCA, ANIT, bosentan and ML-9 inhibited Ca^2+^/CaM-dependent MLCK instead of ROCK and led to BC dilation. (**4**) BDM inhibited myosin II heavy chain ATPase activity and disrupted acto-myosin interaction causing BC dilation. ROCK, Rho-kinase; Ca^2+^, calcium; CaM, calmodulin; MLCK, myosin light chain kinase; MLCP, myosin light chain phosphatase; MLC, myosin light chain; BC, bile canaliculi.

In summary, our work demonstrates that spontaneous rhythmic contractions of BC are essential for bile flow. These contractions are correlated with the rhythm of MLC2 phosphorylation/dephosphorylation. By targeting distinct regulators of the ROCK/MLCK/myosin pathway, cholestatic compounds with diverse chemical structures and therapeutic properties can induce alterations in the contractile movements of BC and impairments in the secretion of BA (Fig. 11). This study highlights new insights into the mechanisms underlying the disruption of BA secretion and identifies new potential predictive biomarkers for drug-induced cholestasis.

## Materials and Methods

### Reagents

Cyclosporine A (CsA), chlorpromazine (CPZ), deoxycholic acid (DCA), 2,3-butanedione monoxime (BDM), 1α-naphthyl isothiocyanate (ANIT), methylthiazoletetrazolium (MTT), 4-(1-Aminoethyl)-N-(4-Pyridyl) cyclohexanecarboxamide dihydrochloride (Y-27632), 1-(5-Chloronaphthalene-1-sulfonyl)-1H-hexahydro-1,4-diazepine hydrochloride (ML-9), taurocholic acid (TA) and 5 (and 6)-carboxy-2,7-dichlorofluorescein diacetate (CDFDA) were purchased from Sigma (St. Quentin Fallavier, France). Rho-associated kinase (ROCK) activity assay Kit was from Merck Millipore (Fontenay sous Bois, France). 3α-hydroxy-7-nitrobenzoxadiazolyl-ursodeoxycholic acid (NBD-UDCA) fluorescent analogue was a gift from Pr A. F. Hoffmann (San Diego, USA). Fasudil (HA-1077) was purchased from BPS Bioscience (Le Perray En Yvelines, France). Bosentan was obtained from Sequoia Research Products (Pangbourne, U.K.). Calmodulin (CaM) was from Merck Chemicals (Fontenay sous Bois, France). Phalloidin fluoprobe was purchased from Interchim (Montluçon, France). [^3^H]-Taurocholic acid ([^3^H]-TA) was from Perkin Elmer (Boston, MA, USA). Specific antibodies against Phospho-Myosin Light Chain 2 (Ser19) (diluted 1:1000, catalog 3675), total MLC2 (diluted 1:1000, catalog 8505) were purchased from Cell Signalling Technology (Schuttersveld, Netherlands). Anti-multidrug-resistance-protein 1 (MDR1) (diluted 1:50, catalog 517310) was obtained from Calbiochem (Saint Aubin, France). Anti-zona occludens protein 1 (ZO-1) (diluted 1:500, catalog 610966) antibody was obtained from BD Biosciences (Le Pont de Claix, France). Anti-MRP2 (1:200, catalog 3373) antibody was from Abcam (Cambridge, UK). Secondary antibodies (diluted 1:500) were purchased from Invitrogen (Saint Aubin, France). Hoechst dye was from Promega (Madison, WI, USA). Other chemicals were of reagent grade.

### Cell cultures

HepaRG cells were seeded at a density of 2.6 × 10^4 ^cells/cm^2^ in Williams’ E medium supplemented with 2 mM glutamax, 100 U/mL penicillin, 100 μg/mL streptomycin, 10% HyClone foetal calf serum, 5 μg/mL insulin, and 50 μM hydrocortisone hemisuccinate. At confluence (after 2 weeks), the HepaRG cells were shifted to the same medium supplemented with 1.7% dimethyl sulfoxide for 2 additional weeks to obtain confluent differentiated cultures containing equal proportions of hepatocyte-like and progenitor/primitive biliary-like cells^39^,^40^. These differentiated hepatic cell cultures were used for the analytical assays.

Human hepatocytes were obtained from Biopredic International (St Gregoire, France), who holds all permits required for the acquisition and transformation of human biological materials to be used in research (AC-2007-43/ law CSP L1245-2/IE-2011-566). Informed consents were obtained from all subjects (Center of Biological Resources, Rennes, France, BB-0033-00,056). Research protocols were performed in accordance with French legal guidelines (French Ministery of Health). All the experimental protocols with human hepatocytes were approved by the local institutional committee (Pontchaillou Hospital, Rennes, France). The hepatocytes were isolated by collagenase perfusion of histologically normal liver fragments from 4 adult donors undergoing resection for primary and secondary tumours. The primary cultures were obtained by hepatocyte seeding at a density of 1.5 × 10^5 ^cells/cm^2^ onto collagen-precoated plates in Williams’ E medium supplemented as detailed above except for the addition of 10% foetal calf serum and 1 μg/ml bovine serum albumin. The medium was discarded 12 h after cell seeding, and the cultures were then maintained in the same medium as for the HepaRG cells and designated as conventional cultured HH (CCHH), or maintained for 24 to 48 h and then washed with cold medium and overlaid with matrigel at a concentration of 0.25 mg/mL in ice-cold Williams’ E medium for the preparation of sandwich cultured HH (SCHH). The medium of both the CCHH and SCHH preparations was renewed daily. The CCHH and SCHH were prepared from the same donors and analysed in parallel. The SCHH cultures were used after at least a 3 day-matrigel overlay.

### Immunolabelling

The cells were washed with warm phosphate buffered saline (PBS), fixed with either methanol for 15 min at −20 °C or with 4% paraformaldehyde for 20 min, and then washed three times with cold PBS. After paraformaldehyde fixation, the cells were permeabilized for 20 min with 0.3% Triton X-100 in PBS followed by a 1 h incubation in PBS containing 1% bovine serum albumin and 5% normal donkey serum. The cells were then incubated overnight with primary antibodies directed against MDR1, MRP2 and ZO-1 and diluted in PBS containing 1% bovine serum albumin and 5% normal donkey serum. After washing with cold PBS, the cells were incubated for 2 h with mouse or rabbit Cy^®^5 -labelled secondary antibodies (excitation/emission: 649/670 nm). Finally, the cells were again washed with cold PBS and incubated with a rhodamine-phalloidin fluoroprobe SR101 (200 U/ml) (excitation/emission: 540/565 nm) diluted at 1/100 for F-actin labelling for 20 min^41^. The nuclei were labelled with 5 ng/ml Hoechst dye (excitation/emission: 346/460 nm). Immunofluorescence images were captured using a Cellomics ArrayScan VTI HCS Reader (Thermo Scientific, Waltham, MA, USA).

### BC area quantification

The BC area quantification was based on ZO-1 protein immunolabelling and phase-contrast images.

#### ZO-1 immunolabelling

After capturing the ZO-1 protein immunofluorescence images, the BC were quantified using the Cell Health Profiling V4 bio application of the ArrayScan software (Thermo Scientific). Segmentation was performed by adjusting the shape, area and brightness parameters to eliminate non-corresponding objects. Based on their areas (labelled objects), the BC were classified into 3 categories: 20–50, 50–100 and >100 μm^2^ (Supplementary Fig. 1). An analysis was performed on 10 images per condition.

#### Phase-contrast images

The mean BC areas were determined from 3 zones per condition using ImageJ 1.48 software at T0 and after 3 h of compound exposure. Briefly, after capturing the time-lapse images, bright objects corresponding to BC were segmented by adjusting the shape and area parameters to exclude non-corresponding objects. The data are presented as the fold change in the BC mean area after 3 h relative to their mean area at T0.

### Electron microscopy

HepaRG cells were fixed by the addition of 2.5% glutaraldehyde for 30 min. After fixation, the specimens were rinsed with 0.2 M Na cacodylate buffer and post-fixed with 2% osmium tetroxide for 30 min. After further rinsing, the samples were dehydrated, infiltrated with a mixture of acetone-eponate (50/50) and embedded in DMP30-Eponate. Ultrathin sections were examined with a JEOL 100CX II electron microscope^42^.

### Western blotting analysis of MLC2 phosphorylation

The HepaRG cells were treated with the 6 tested compounds for different time points (0–225 min), washed with cold PBS, and finally suspended in cell lysis buffer supplemented with protease and phosphatase inhibitors (Roche, Mannheim, Germany). Aliquots containing an equivalent total protein content as determined by the Bradford procedure with bovine serum albumin as the standard were subjected to sodium dodecyl sulfate/12% polyacrylamide gel electrophoresis, electrotransferred to Immobilon-P membranes, and incubated overnight with primary antibodies directed against p-MLC2 and total MLC2 (dilution: 1/1000). After exposure to a horseradish peroxidase conjugated anti-mouse/rabbit antibody (dilution: 1/5000) (Dako, Les Ulis, France), the membranes were incubated with a chemiluminescence reagent (Millipore, Billerica, MA, USA) and the bands were then visualized with Fusion-Capt software (Vilber Lourmat, Marne La Vallée, France) and quantified using ImageJ 1.48.

### CDF clearance

The cells were washed with warm Williams’ E medium without phenol red and incubated in 3 μM CDFDA for 30 min at 37 °C in the same medium used for passive intracellular accumulation. Upon hydrolysis by the intracellular esterases, CDFDA was converted to fluorescent CDF (excitation/emission: 488/509 nm) and directed towards the biliary pole by membrane transporters, particularly MRP2. Then, after washing, the cells were treated with the 6 compounds for 2 and 3 h and imaging was performed using a Cellomics ArrayScan VTI HCS Reader (Thermo Scientific).

### NBD-UDCA clearance

The cells were washed with warm Williams’ E medium without phenol red and incubated in 5 μM NBD-UDCA (excitation/emission: 488/509 nm) for 30 min at 37 °C in Williams’ E medium without phenol red. NBD-UDCA is secreted into BC by membrane transporters, mainly BSEP^43^. Then, after washing, the cells were treated with the 6 compounds for 2 h and imaging was performed using an inverted microscope (Zeiss Axiovert 200M and AxioCam MRm).

### Taurocholate acid clearance

To investigate the effect of the compounds on TA clearance from the cell layer, the cells were first exposed to 43.3 nM [^3^H]-TA for 30 min to induce its intracellular accumulation, washed with standard buffer and then incubated with the tested compounds for 2 h in a standard buffer containing Ca^2+^ and Mg^2+^. After incubation, the cells were washed and scraped in 0.1 N NaOH, and the remaining radiolabelled substrate was measured through scintillation. [^3^H]-TA clearance was determined based on its accumulation in the cell layers (cells + BC) and calculated relative to the control using the following formula: [^3^H]-TA clearance = [^3^H]-TA accumulation in (cells + BC)_*Control*_/[^3^H]-TA accumulation in cell layer_*Tested compound*_  * 100.

### Time-lapse cell imaging

Phase-contrast images of the HepaRG cells, CCHH and SCHH were captured every minute using time-lapse phase-contrast videomicroscopy. An inverted microscope (Zeiss Axiovert 200 M) was equipped with a thermostatic chamber (37 °C and 5% CO_2_) to maintain the cells under normal culture conditions. The images were captured with an AxioCam MRm camera.

### BC dynamics analysis

Contraction/relaxation activity and the opening/closing rhythm of BC (spikes) were evaluated by time-lapse cell imaging and quantified by a software video analysis and a modelling tool (Tracker 4.87). Briefly, circular frames delimiting S-BC and the connections S-BC/T-BC were manually designed using the free hand selection tool of Tracker 4.87 to track and record brightness variations. These records reflected the area of S-BC and spikes at the connections between S-BC and T-BC (Supplementary Fig. 2).

### ROCK activity

ROCK activity was measured with a Rho-associated kinase activity assay Kit (Millipore, catalogue CSA001) according to the manufacturer’s protocol with certain modifications. Briefly, HepaRG cells were treated with the tested compounds. After 1 h, the cells were lysed with a lysis buffer supplemented with anti-protease, and then 50 μL of the lysate was deposited in 96-well multi-strip plates pre-coated with MYPT1 supplied with 10 mM DTT, 2 mM MgCl2 and 10 mM ATP for 60 min at 30 °C. An anti-phospho-MYPT1 (Thr696) antibody was added for 1 h, and then goat anti-rabbit IgG HRP secondary antibody was added for another 1 h, and chromogenic substrate tetra-methylbenzidine (TMB) was added for 15 min. Absorbance at 450 nm reflected the relative amount of ROCK activity in the sample, which was evaluated relative to the total protein content of each sample.

### Cell viability

Cytotoxicity was evaluated by the MTT colorimetric assay. Briefly, the cells were seeded in 96-well plates and treated with various concentrations of the compounds in triplicate for 4 h. After removing the medium, 100 μl of serum-free medium containing MTT (0.5 mg/ml) was added to each well and incubated for 2 h at 37 °C. The water-insoluble formazan was dissolved in 100 μl dimethyl sulfoxide, and absorbance was measured at 550 nm.

### Statistical analysis

A one-way ANOVA with a multiple comparison test (GraphPad Prism 5.00) was performed to compare the data. Each value corresponded to the mean ± SEM of three independent experiments. Data were considered significantly different at p < 0.05.

## Additional Information

**How to cite this article**: Sharanek, A. *et al.* Rho-kinase/myosin light chain kinase pathway plays a key role in the impairment of bile canaliculi dynamics induced by cholestatic drugs. *Sci. Rep.* **6**, 24709; doi: 10.1038/srep24709 (2016).

## Supplementary Material

Supplementary Information

Supplementary Video 1

Supplementary Video 2

Supplementary Video 3

Supplementary Video 4

Supplementary Video 5

## Figures and Tables

**Figure 1:**
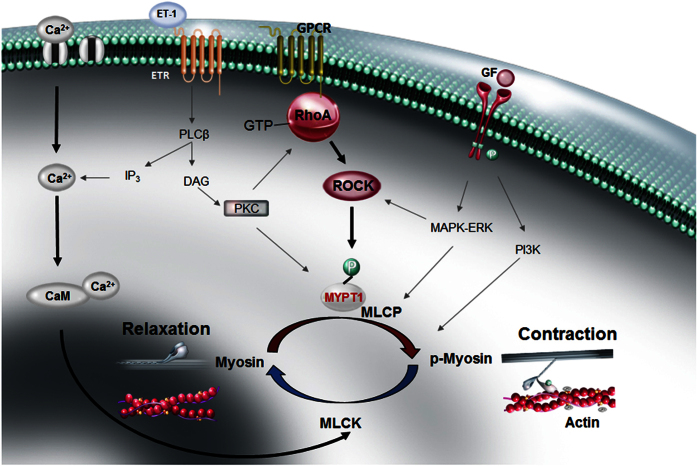
Schematic representation of MLC phosphorylation regulation by Rho-kinase and myosin light chain kinase. ROCK, Rho-kinase; Ca^2+^, calcium; CaM, calmodulin; MLCK, myosin light chain kinase; MLCP, myosin light chain phosphatase; MYPT-1, myosin phosphatase target subunit 1; MLC, myosin light chain, ET-1, endothelin-1; ETR, endothelin receptor; IP3, inositol 1,4,5-triphosphate; GPCR, G-protein coupled receptor; PKC, protein kinase C; PLCβ, phospholipase C β; DAG, diacylglycerol; PI3K, phosphatidylinositol 3-kinases ; ERK, extracellular signal-regulated kinases; MAPK, mitogen-activated protein kinases ; GF, growth factor.

## References

[b1] Padda M. S., Sanchez M., Akhtar A. J. & Boyer J. L. Drug-induced cholestasis. Hepatology 53, 1377–1387 (2011). 21480339 10.1002/hep.24229 PMC3089004

[b2] Bjornsson E. S. & Jonasson J. G. Drug-induced cholestasis. Clin Liver Dis 17, 191–209 (2013). 23540497 10.1016/j.cld.2012.11.002

[b3] Morgan R. E. *et al.* A multifactorial approach to hepatobiliary transporter assessment enables improved therapeutic compound development. Toxicol Sci 136, 216–241 (2013). 23956101 10.1093/toxsci/kft176

[b4] Stieger B. Role of the bile salt export pump, BSEP, in acquired forms of cholestasis. Drug Metab Rev 42, 437–445 (2010). 20028269 10.3109/03602530903492004

[b5] Welch M. A., Kock K., Urban T. J., Brouwer K. L. & Swaan P. W. Toward predicting drug-induced liver injury: parallel computational approaches to identify multidrug resistance protein 4 and bile salt export pump inhibitors. Drug Metab Dispos 43, 725–734 (2015). 25735837 10.1124/dmd.114.062539 PMC4407708

[b6] Wakabayashi Y., Dutt P., Lippincott-Schwartz J. & Arias I. M. Rab11a and myosin Vb are required for bile canalicular formation in WIF-B9 cells. Proc Natl Acad Sci USA 102, 15087–15092 (2005). 16214890 10.1073/pnas.0503702102 PMC1257697

[b7] Nossaman B. D. & Kadowitz P. J. The role of the RhoA/rho-kinase pathway in pulmonary hypertension. Curr Drug Discov Technol 6, 59–71 (2009). 19275543 10.2174/157016309787581057

[b8] Lee D. L., Webb R. C. & Jin L. Hypertension and RhoA/Rho-kinase signaling in the vasculature: highlights from the recent literature. Hypertension 44, 796–799 (2004). 15520302 10.1161/01.HYP.0000148303.98066.ab

[b9] Mills J. C., Stone N. L., Erhardt J. & Pittman R. N. Apoptotic membrane blebbing is regulated by myosin light chain phosphorylation. J Cell Biol 140, 627–636 (1998). 9456322 10.1083/jcb.140.3.627 PMC2140178

[b10] Isotani E. *et al.* Real-time evaluation of myosin light chain kinase activation in smooth muscle tissues from a transgenic calmodulin-biosensor mouse. Proc Natl Acad Sci USA 101, 6279–6284 (2004). 15071183 10.1073/pnas.0308742101 PMC395960

[b11] Herrema H. *et al.* Rho kinase, myosin-II, and p42/44 MAPK control extracellular matrix-mediated apical bile canalicular lumen morphogenesis in HepG2 cells. Mol Biol Cell 17, 3291–3303 (2006). 16687572 10.1091/mbc.E06-01-0067 PMC1552049

[b12] Fu D., Wakabayashi Y., Lippincott-Schwartz J. & Arias I. M. Bile acid stimulates hepatocyte polarization through a cAMP-Epac-MEK-LKB1-AMPK pathway. Proc Natl Acad Sci USA 108, 1403–1408 (2011). 21220320 10.1073/pnas.1018376108 PMC3029747

[b13] Homolya L. *et al.* LKB1/AMPK and PKA control ABCB11 trafficking and polarization in hepatocytes. Plos One 9, e91921 (2014). 24643070 10.1371/journal.pone.0091921 PMC3958433

[b14] Fu D. *et al.* Coordinated elevation of mitochondrial oxidative phosphorylation and autophagy help drive hepatocyte polarization. Proc Natl Acad Sci USA 110, 7288–7293 (2013). 23589864 10.1073/pnas.1304285110 PMC3645550

[b15] Zhou Q. *et al.* Intrahepatic upregulation of RhoA and Rho-kinase signalling contributes to increased hepatic vascular resistance in rats with secondary biliary cirrhosis. Gut 55, 1296–1305 (2006). 16492715 10.1136/gut.2005.081059 PMC1860046

[b16] Arias I. M. *et al.* The biology of the bile canaliculus, 1993. Hepatology 17, 318–329 (1993). 8428731

[b17] LeCluyse E. L., Audus K. L. & Hochman J. H. Formation of extensive canalicular networks by rat hepatocytes cultured in collagen-sandwich configuration. Am J Physiol 266, C1764–1774 (1994). 8023906 10.1152/ajpcell.1994.266.6.C1764

[b18] Antherieu S. *et al.* Oxidative stress plays a major role in chlorpromazine-induced cholestasis in human HepaRG cells. Hepatology 57, 1518–1529 (2013). 23175273 10.1002/hep.26160

[b19] Sharanek A. *et al.* Different dose-dependent mechanisms are involved in early cyclosporine a-induced cholestatic effects in hepaRG cells. Toxicol Sci 141, 244–253 (2014). 24973091 10.1093/toxsci/kfu122 PMC4833109

[b20] Sharanek A. *et al.* Cellular Accumulation and Toxic Effects of Bile Acids in Cyclosporine A-Treated HepaRG Hepatocytes. Toxicol Sci 147, 573–587 (2015). 26198044 10.1093/toxsci/kfv155

[b21] Orsler D. J., Ahmed-Choudhury J., Chipman J. K., Hammond T. & Coleman R. ANIT-induced disruption of biliary function in rat hepatocyte couplets. Toxicol Sci 47, 203–210 (1999). 10220858 10.1093/toxsci/47.2.203

[b22] Barnwell S. G., Tuchweber B. & Yousef I. M. Biliary lipid secretion in the rat during infusion of increasing doses of unconjugated bile acids. Biochim Biophys Acta 922, 221–233 (1987). 3676344 10.1016/0005-2760(87)90158-5

[b23] Miyairi M. *et al.* Taurocholate accelerates bile canalicular contractions in isolated rat hepatocytes. Gastroenterology 87, 788–792 (1984). 6468869

[b24] Watanabe T., Hosoya H. & Yonemura S. Regulation of myosin II dynamics by phosphorylation and dephosphorylation of its light chain in epithelial cells. Mol Biol Cell 18, 605–616 (2007). 17151359 10.1091/mbc.E06-07-0590 PMC1783795

[b25] Morgan R. E. *et al.* Interference with bile salt export pump function is a susceptibility factor for human liver injury in drug development. Toxicol Sci 118, 485–500 (2010). 20829430 10.1093/toxsci/kfq269

[b26] Dawson S., Stahl S., Paul N., Barber J. & Kenna J. G. *In vitro* inhibition of the bile salt export pump correlates with risk of cholestatic drug-induced liver injury in humans. Drug Metab Dispos 40, 130–138 (2012). 21965623 10.1124/dmd.111.040758

[b27] Pedersen J. M. *et al.* Early identification of clinically relevant drug interactions with the human bile salt export pump (BSEP/ABCB11). Toxicol Sci 136, 328–343 (2013). 24014644 10.1093/toxsci/kft197 PMC3858191

[b28] Rodrigues A. D. *et al.* Drug-induced perturbations of the bile acid pool, cholestasis, and hepatotoxicity: mechanistic considerations beyond the direct inhibition of the bile salt export pump. Drug Metab Dispos 42, 566–574 (2014). 24115749 10.1124/dmd.113.054205

[b29] Wood R. L. An Electron Microscope Study of Developing Bile Canaliculi in the Rat. Anat Rec 151, 507–529 (1965). 14326981 10.1002/ar.1091510403

[b30] Fu D., Wakabayashi Y., Ido Y., Lippincott-Schwartz J. & Arias I. M. Regulation of bile canalicular network formation and maintenance by AMP-activated protein kinase and LKB1. J Cell Sci 123, 3294–3302 (2010). 20826460 10.1242/jcs.068098 PMC2939801

[b31] Bachour-El Azzi P. *et al.* Comparative Localization and Functional Activity of the Main Hepatobiliary Transporters in HepaRG Cells and Primary Human Hepatocytes. Toxicol Sci 145, 157–168 (2015). 25690737 10.1093/toxsci/kfv041 PMC4833040

[b32] Oshio C. & Phillips M. J. Contractility of bile canaliculi: implications for liver function. Science 212, 1041–1042 (1981). 7015506 10.1126/science.7015506

[b33] Watanabe N., Tsukada N., Smith C. R. & Phillips M. J. Motility of bile canaliculi in the living animal: implications for bile flow. J Cell Biol 113, 1069–1080 (1991). 2040644 10.1083/jcb.113.5.1069 PMC2289005

[b34] Adelstein R. S., Pato M. D., Sellers J. R., de Lanerolle P. & Conti M. A. Regulation of actin-myosin interaction by reversible phosphorylation of myosin and myosin kinase. Cold Spring Harb Symp Quant Biol 46 Pt 2, 921–928 (1982). 6125296 10.1101/sqb.1982.046.01.086

[b35] Watanabe N. *et al.* Taurolithocholate impairs bile canalicular motility and canalicular bile secretion in isolated rat hepatocyte couplets. World J Gastroenterol 12, 5320–5325 (2006). 16981261 10.3748/wjg.v12.i33.5320 PMC4088198

[b36] Arikawa K., Hicks J. L. & Williams D. S. Identification of actin filaments in the rhabdomeral microvilli of Drosophila photoreceptors. J Cell Biol 110, 1993–1998 (1990). 2112548 10.1083/jcb.110.6.1993 PMC2116135

[b37] Kamm K. E. & Stull J. T. Dedicated myosin light chain kinases with diverse cellular functions. J Biol Chem 276, 4527–4530 (2001). 11096123 10.1074/jbc.R000028200

[b38] Somlyo A. P. & Somlyo A. V. Ca^2+^ sensitivity of smooth muscle and nonmuscle myosin II: modulated by G proteins, kinases, and myosin phosphatase. Physiol Rev 83, 1325–1358 (2003). 14506307 10.1152/physrev.00023.2003

[b39] Cerec V. *et al.* Transdifferentiation of hepatocyte-like cells from the human hepatoma HepaRG cell line through bipotent progenitor. Hepatology 45, 957–967 (2007). 17393521 10.1002/hep.21536

[b40] Gripon P. *et al.* Infection of a human hepatoma cell line by hepatitis B virus. Proc Natl Acad Sci USA 99, 15655–15660 (2002). 12432097 10.1073/pnas.232137699 PMC137772

[b41] Pernelle K. *et al.* Automated detection of hepatotoxic compounds in human hepatocytes using HepaRG cells and image-based analysis of mitochondrial dysfunction with JC-1 dye. Toxicol Appl Pharmacol 254, 256–266 (2011). 21569786 10.1016/j.taap.2011.04.018

[b42] Antherieu S., Rogue A., Fromenty B., Guillouzo A. & Robin M. A. Induction of vesicular steatosis by amiodarone and tetracycline is associated with up-regulation of lipogenic genes in HepaRG cells. Hepatology 53, 1895–1905 (2011). 21391224 10.1002/hep.24290

[b43] Wang R. *et al.* Defective canalicular transport and toxicity of dietary ursodeoxycholic acid in the abcb11−/− mouse: transport and gene expression studies. Am J Physiol Gastrointest Liver Physiol 305, G286–294 (2013). 23764895 10.1152/ajpgi.00082.2013

