# Supplementary material for: Rho-kinase/myosin light chain kinase pathway plays a key role in the impairment of bile canaliculi dynamics induced by cholestatic drugs
Source: Sci Rep. 2016 May 12;6:24709. doi: 10.1038/srep24709 (PMC4867683; doi:10.1038/srep24709)
Supplement: Supplementary Information [file srep24709-s1.pdf]

Supplementary information

Rho-kinase/myosin light chain kinase pathway plays a key role in the impairment of bile canaliculi dynamics induced by cholestatic drugs

Ahmad Sharanek[1,2*], Audrey Burban[1,2*], Matthew Burbank[1,2], Rémy Le Guevel[3], Ruoya Li[4], André Guillouzo[1,2] and Christiane Guguen-Guillouzo[1,2,4†]

[1]INSERM U991, Liver Metabolisms and Cancer, Rennes, France,

[2]Rennes 1 University, France,

[3]ImPACcell platform, Biosit, Rennes 1 University, France

[4]Biopredic International, St Grégoire, France.

*** Both authors contributed equally to this work**

[†]Correspondence should be addressed at INSERM UMR 991, Université de Rennes 1, Faculté de Pharmacie, F-35043 Rennes cedex, France. Tel.: (33).2.23.23.53.51; christiane.guillouzo@univ-rennes1.fr (C. Guguen-Guillouzo).

Supplementary Table 1. Characteristics of the 6 tested compounds

	Molecules	Therapeutic use	Cmax (µM)	*In vivo* hepatotoxicity	*In vitro* hepatotoxicity	References
Drugs	Chlorpromazine (CPZ)	Antipsychotic	0.1–0.2	• Intrahepatic cholestasis<br>• Bile duct vanishing syndrome<br>• Hepatocellular Necrosis<br>• Phospholipidosis	• Oxidative stress<br>• Bile flow inhibition<br>• Alteration of mitochondrial membrane potential<br>• Bile canaliculi constriction	[1-3]
	Cyclosporine A (CsA)	Immunosup-pressant	1.15	• Cholestatic jaundice<br>• hyperbilirubinemia<br>• Elevated transaminases	• Oxidative stress<br>• BSEP, MRP2, MDR1 inhibition<br>• Inhibition of bile secretion<br>• Bile canaliculi constriction	[4-9]
	Bosentan	Antipulmonary artery hypertension	6.75	• Elevated ASAT/ALAT (3 x/20x)<br>• Increased serum bile acids	• BSEP inhibition	[10]
	Fasudil	Antipulmonary artery hypertension	0.820	-	-	-
Other compounds	ANIT (Alpha-naphthylisothio cyanate)	Cholestatic model compound	-	• Cholestasis<br>• Hyperbilirubinemia<br>• Necrosis of bile duct epithelial cells<br>• Cholangiolitic hepatitis	• Increased serum bilirubin, ASAT and ALAT	[11-14]
	Deoxycholic acid (DCA)	Secondary bile acid	3-7	• Increased serum transaminases activities<br>• Increased total bile acids and bilirubin concentrations in serum	• Cell lysis<br>• Intracellular ROS generation increase<br>• Mitochondrial instability in hepatocytes	[15-20]

Therapeutic use, Cmax, and known hepatotoxicity of the tested compounds.

References to Table 1

1 Anthérieu, S. *et al.* Oxidative stress plays a major role in chlorpromazine-induced cholestasis in human HepaRG cells. *Hepatology* **57**, 1518-1529, (2013).

2 Velayudham, L. S. & Farrell, G. C. Drug-induced cholestasis. *Expert Opin Drug Saf* **2**, 287-304, (2003).

3 Padda, M. S., Sanchez, M., Akhtar, A. J. & Boyer, J. L. Drug-induced cholestasis. *Hepatology* **53**, 1377-1387, (2011).

4 Hagar, H. H. The protective effect of taurine against cyclosporine A-induced oxidative stress and hepatotoxicity in rats. *Toxicol Lett* **151**, 335-343, (2004).
5 Sharanek, A. *et al.* Different dose-dependent mechanisms are involved in early cyclosporine a-induced cholestatic effects in hepaRG cells. *Toxicol Sci* **141**, 244-253, (2014).
6 Roman, I. D. & Coleman, R. Disruption of canalicular function in isolated rat hepatocyte couplets caused by cyclosporin A. *Biochem Pharmacol* **48**, 2181-2188, (1994).
7 Princen, H. M., Meijer, P., Wolthers, B. G., Vonk, R. J. & Kuipers, F. Cyclosporin A blocks bile acid synthesis in cultured hepatocytes by specific inhibition of chenodeoxycholic acid synthesis. *Biochem J* **275 (Pt 2)**, 501-505, (1991).
8 Mizuta, K. *et al.* Cyclosporine inhibits transport of bile acid in rats: comparison of bile acid composition between liver and bile. *Transplant Proc* **31**, 2755-2756, (1999).
9 Whiting, P. H., Thomson, K. J., Saunders, N. J. & Simpson, J. G. Cyclosporin A nephrotoxicity in streptozotocin-diabetic rats. *Transplant Proc* **21**, 946-947, (1989).
10 Fattinger, K. *et al.* The endothelin antagonist bosentan inhibits the canalicular bile salt export pump: a potential mechanism for hepatic adverse reactions. *Clin Pharmacol Ther* **69**, 223-231, (2001).
11 Hill, D. A., Jean, P. A. & Roth, R. A. Bile duct epithelial cells exposed to alpha-naphthylisothiocyanate produce a factor that causes neutrophil-dependent hepatocellular injury in vitro. *Toxicol Sci* **47**, 118-125, (1999).
12 Roos, F., Terrell, T. G., Godowski, P. J., Chamow, S. M. & Schwall, R. H. Reduction of alpha-naphthylisothiocyanate-induced hepatotoxicity by recombinant human hepatocyte growth factor. *Endocrinology* **131**, 2540-2544, (1992).
13 Dahm, L. J., Bailie, M. B. & Roth, R. A. Relationship between alpha-naphthylisothiocyanate-induced liver injury and elevations in hepatic non-protein sulfhydryl content. *Biochem Pharmacol* **42**, 1189-1194, (1991).
14 Orsler, D. J., Ahmed-Choudhury, J., Chipman, J. K., Hammond, T. & Coleman, R. ANIT-induced disruption of biliary function in rat hepatocyte couplets. *Toxicol Sci* **47**, 203-210, (1999).

15 Song, P., Zhang, Y. & Klaassen, C. D. Dose-response of five bile acids on serum and liver bile Acid concentrations and hepatotoxicty in mice. *Toxicol Sci* **123**, 359-367, (2011).

16 Delzenne, N. M., Calderon, P. B., Taper, H. S. & Roberfroid, M. B. Comparative hepatotoxicity of cholic acid, deoxycholic acid and lithocholic acid in the rat: in vivo and in vitro studies. *Toxicol Lett* **61**, 291-304, (1992).

17 Sokol, R. J., Winklhofer-Roob, B. M., Devereaux, M. W. & McKim, J. M., Jr. Generation of hydroperoxides in isolated rat hepatocytes and hepatic mitochondria exposed to hydrophobic bile acids. *Gastroenterology* **109**, 1249-1256, (1995).

18 Rodrigues, C. M., Fan, G., Wong, P. Y., Kren, B. T. & Steer, C. J. Ursodeoxycholic acid may inhibit deoxycholic acid-induced apoptosis by modulating mitochondrial transmembrane potential and reactive oxygen species production. *Mol Med* **4**, 165-178, (1998).

19 Qiao, L. *et al.* Deoxycholic acid (DCA) causes ligand-independent activation of epidermal growth factor receptor (EGFR) and FAS receptor in primary hepatocytes: inhibition of EGFR/mitogen-activated protein kinase-signaling module enhances DCA-induced apoptosis. *Mol Biol Cell* **12**, 2629-2645, (2001).

20 Zucchini-Pascal, N., de Sousa, G., Pizzol, J. & Rahmani, R. Pregnane X receptor activation protects rat hepatocytes against deoxycholic acid-induced apoptosis. *Liver Int* **30**, 284-297, (2010).

A) Nuclei **B)** ZO-1 **C)** BC segmentation

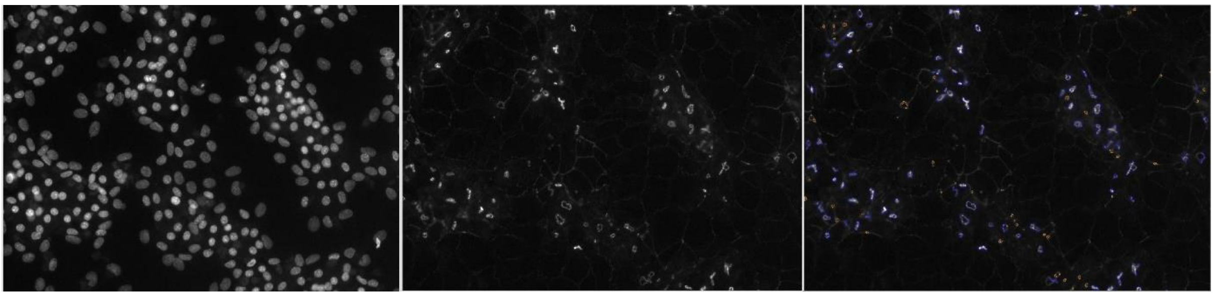

Supplementary Fig. 1. BC area quantification. The BC area quantification was based on ZO-1 protein immunolabelling using the Cell Health Profiling V4 bio application of the ArrayScan software (Thermo Scientific). **A)** Hoechst-labelled nuclei. **B)** ZO-1 protein immunofluorescence images. **C)** Segmentation was performed by adjusting the shape, area and brightness parameters to eliminate non-corresponding objects. An analysis was performed on 10 images per condition.

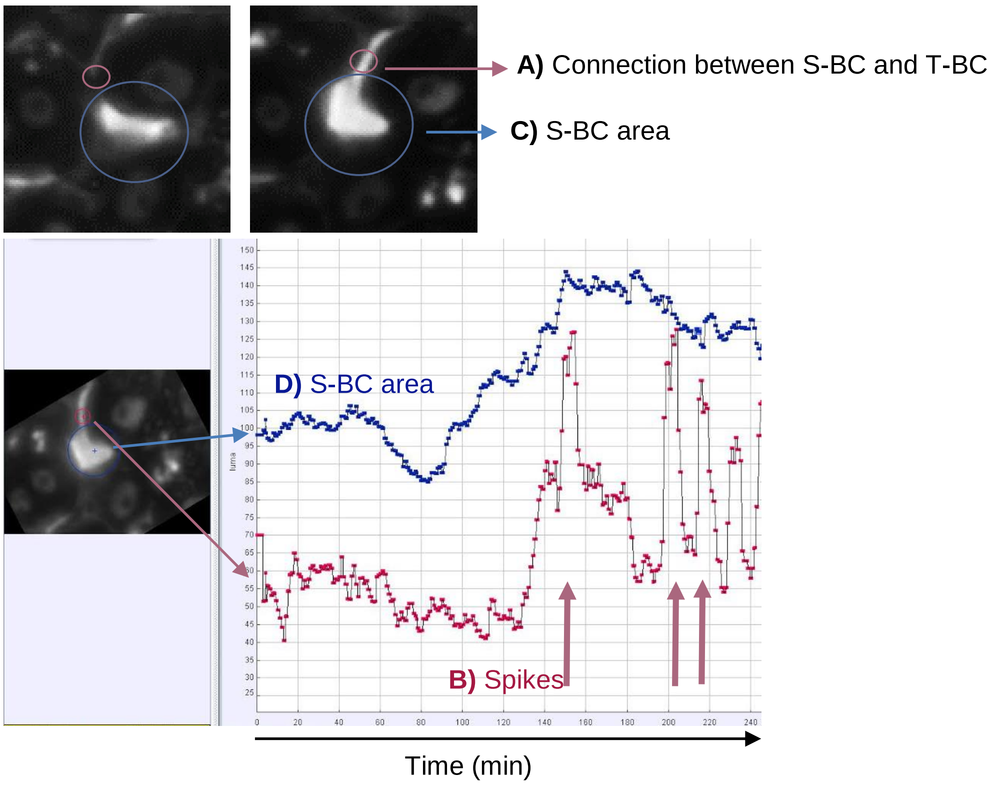

Supplementary Fig. 2. BC dynamics analysis. Contraction/relaxation activity and the opening/closing rhythm of BC (spikes) were evaluated by time-lapse cell imaging and quantified by a software video analysis and a modelling tool (Tracker 4.87). **A, B)** Circular frames (red) delimiting the connections between S-BC and T-BC to track brightness variations and record the rhythm of spikes (red graph). **C, D)** Circular frames (blue) delimiting S-BC to record changes in the area of S-BC (blue graph).

Supplementary Video legends

Supplementary Video 1. Rhythmic dynamic movements of BC in HepaRG cells. Images were captured each minute during 4 h under time-lapse phase-contrast videomicroscopy equipped with a thermostatic chamber (37°C and CO_2) to maintain the cells under physiological conditions. Video is accelerated 600x.

Supplementary Video 2. Rhythmic dynamic movements of BC in CCHH. Images were captured each minute during 4 h under time-lapse phase-contrast videomicroscopy equipped with a thermostatic chamber (37°C and CO_2) to maintain the cells under physiological conditions. Video is accelerated 600x.

Supplementary Video 3. Rhythmic dynamic movements of BC in SCHH. Images were captured each minute during 4 h under time-lapse phase-contrast videomicroscopy equipped with a thermostatic chamber (37°C and CO_2) to maintain the cells under physiological conditions. Video is accelerated 600x.

Supplementary Video 4. Constriction of BC and loss of their rhythmic dynamics in CPZ-treated HepaRG cells. Images of 50µM CPZ-treated cells were captured each minute during 4 h under time-lapse phase-contrast videomicroscopy equipped with a thermostatic chamber (37°C and CO_2). Video is accelerated 600x.

Supplementary Video 5. Dilation of BC and loss of their rhythmic dynamics in fasudil-treated HepaRG cells. Images of 50µM fasudil-treated cells were captured each minute during 4 h under time-lapse phase-contrast videomicroscopy equipped with a thermostatic chamber (37°C and CO_2). Video is accelerated 600x.
